# 6,8-Diprenylorobol Induces Apoptosis in Human Hepatocellular Carcinoma Cells via Activation of FOXO3 and Inhibition of CYP2J2

**DOI:** 10.1155/2020/8887251

**Published:** 2020-11-19

**Authors:** Chang Min Lee, Jongsung Lee, Su-Nyeong Jang, Jong Cheol Shon, Zhexue Wu, Kyungmoon Park, Kwang-Hyeon Liu, See-Hyoung Park

**Affiliations:** ^1^Department of Bio and Chemical Engineering, Hongik University, Sejong 30016, Republic of Korea; ^2^Department of Integrative Biotechnology, Sungkyunkwan University, Suwon 16419, Republic of Korea; ^3^BK21 Plus KNU Multi-Omics Based Creative Drug Research Team, College of Pharmacy and Research Institute of Pharmaceutical Sciences, Kyungpook National University, Daegu 41566, Republic of Korea; ^4^Environmental Chemistry Research Center, Korea Institute of Toxicology, Jinju 52834, Republic of Korea

## Abstract

6,8-Diprenylorobol is a phytochemical derived from the roots of *Glycyrrhiza uralensis* Fisch. 6,8-Diprenylorobol exhibits several biological activities, but the effects of 6,8-diprenylorobol on cancers have been hardly investigated. This study is aimed at elucidating the anticancer effect and working mechanism of 6,8-diprenylorobol in HepG2 and Huh-7, two kinds of human hepatocellular carcinoma (HCC) cell lines. WST-1, cell counting, and colony formation assays and morphological change analysis showed that 6,8-diprenylorobol treatment decreased the cell viability and proliferation rate. Cell cycle analysis indicated that 6,8-diprenylorobol treatment increased the population of the G1/0 stage. Annexin V/PI double staining and TUNEL analysis showed that 6,8-diprenylorobol treatment increased the apoptotic cell population and DNA fragmentation. Western blot analysis showed that 6,8-diprenylorobol treatment increased the expression of cleaved PARP1, cleaved caspase-3, FOXO3, Bax, Bim, p21, and p27 but decreased the expression of Bcl2 and BclXL. Interestingly, 6,8-diprenylorobol inhibited CYP2J2-mediated astemizole *O*-demethylation and ebastine hydroxylase activities with *K*_*i*_ values of 9.46 and 2.61 *μ*M, respectively. CYP2J2 siRNA transfection enhanced the anticancer effect of 6,8-diprenylorobol in HepG2 and Huh-7 cells through the downregulation of CYP2J2 protein expression and upregulation of FOXO3. Taken together, this study proposes that 6,8-diprenylorobol treatment may be a useful therapeutic option against HCC by targeting CYP2J2 and FOXO3.

## 1. Introduction

In 2000, liver cancer accounts for the ninth leading cause of cancer death but increased to sixth in 2016 [1]. Liver cancer has been recognized as highly fatal, and death rates are increasing much faster than those for any other cancers in the United States [2, 3]. Globally, liver cancer is the second leading cause of death related to cancer [4]. Furthermore, according to recent cancer statistics, liver cancer incidence has increased much faster than any other cancers in both sexes [5]. So far, liver transplantation and resection have been recognized as the most effective treatment for hepatocellular carcinoma (HCC), which is one of the most common liver cancers [6]. However, there are several side effects of these therapeutic methods. After liver transplantation, patients should take medications for the rest of their life to help prevent their body from rejecting the donor's liver [7]. These antirejection medications can cause a variety of side effects, such as bone thinning, diabetes, high blood pressure, and high cholesterol level [8]. Therefore, it is necessary to develop alternative therapeutic strategies to treat HCC.

Phytochemicals are natural compounds produced by many kinds of plants, and the function is generally to support them thrive or thwart predators or pathogens [9]. It is reported that there are several beneficial effects of phytochemicals to health, such as reducing reactive oxygen species (ROS) in the human body [10]. Furthermore, phytochemicals are potential modulators of immunological processes related to anticancer, antioxidant, and anti-inflammatory [11]. For the last ten years, phytochemicals have been widely investigated to develop effective medicine for cancer treatment because phytochemicals have a potential to be developed as an anticancer agents with high efficacy and few side effects [12]. Recent studies showed that phytochemicals are potent modulators of autophagy for cancer treatment [13, 14]. Furthermore, phytochemicals suppressed migration of metastatic breast cancer cells [15].

Cytochrome P450 2J2 (CYP2J2) is a member of the cytochrome P450 enzyme superfamily [16]. CYP2J2 is expressed in the vascular endothelium and is a prominent enzyme modulating metabolism of endogenous polyunsaturated fatty acids [17]. CYP2J2 has been recognized as a crucial biomarker of the disease. According to a recent study, CYP2J2 is a key enzyme in bioactivation of cyclophosphamide and a promising biomarker for hematological malignancies [18]. Interestingly, CYP2J2 is found to be upregulated in various cancers, and it plays a crucial role in cancer cell proliferation and human cancer metastasis [19–21]. Upregulation of let-7b suppressed the expression of CYP2J2 protein in cancerous tissues, which causes the inhibition of tumor phenotypes [22]. Furthermore, CYP2J2 has a protective effect in breast cancer MDA-MB-468 cells against cell death mediated by reactive oxygen species (ROS) [23]. Therefore, CYP2J2 may be an important biomarker to develop anticancer drugs.

Forkhead box O3 (FOXO3) is a member of the O subclass of the forkhead family, and it functions as a transcription factor regulating multiple physiological processes such as programmed cell death, cell cycle, and oxidative stress response [24]. FOXO3 is associated with various diseases, particularly in malignancy of various cancers such as breast, liver, colon, and prostate cancer [25–27]. Specifically, previous studies showed that FOXO3 plays an important role in the regulation of the cancer proliferation and apoptosis process [28, 29]. It is reported that activation of FOXO3 activity inhibits the proliferation of colon cancer HT-29 cells [30]. Moreover, overexpression of FOXO3 induces apoptosis in the human prostate cancer cell line [28]. In addition, activation of FOXO3 displays an anticancer effect on human ovarian cancer SKOV3 cells [31]. Therefore, FOXO3 may be an important therapeutic target of various cancers.

In modern medication, natural compounds have been recognized as an important source of many kinds of drugs. In particular, plant-derived compounds are recognized as a crucial source of useful anticancer agents such as vinblastine, vincristine, and paclitaxel [32]. 6,8-Diprenylorobol is a phytochemical derived from the roots of *Glycyrrhiza uralensis* Fisch [33]. It is reported that 6,8-diprenylorobol has an anti-*Helicobacter pylori* effect and antiestrogenic activity [34, 35]. However, the effects of this phytochemical on cancers have been hardly investigated. The aim of this study is to investigate the anticancer effect of 6,8-diprenylorobol on human hepatocellular carcinoma Huh-7 and HepG2 cells.

## 2. Materials and Methods

### 2.1. Reagents

6,8-Diprenylorobol was purchased from ChemFaces (CheCheng Rd. WETDZ, Wuhan, China) and dissolved in DMSO (Sigma, St. Louis, MO, USA). A 40 mM stock solution of 6,8-diprenylorobol was stored at -20°C. Glucose-6-phosphate (G6P), G6P dehydrogenase, trimipramine, *β*-nicotinamide dinucleotide phosphate (NADP^+^), and mouse anti-*β*-actin antibody (1 : 5000 dilution) were purchased from Sigma. Rabbit anti-FOXO3 (1 : 1000 dilution), rabbit anti-CYP2J2 (1 : 1000 dilution), rabbit anti-p-p38 (1 : 1000 dilution), rabbit anti-p38 (1 : 1000 dilution), rabbit anti-*γ*H2AX (1 : 1000 dilution), rabbit anti-H2AX (1 : 1000 dilution), rabbit anti-p-AKT (1 : 1000 dilution), rabbit anti-AKT (1 : 1000 dilution), rabbit anti-p-ERK (1 : 1000 dilution), rabbit anti-ERK (1 : 1000 dilution), rabbit anti-p-JNK (1 : 1000 dilution), and rabbit anti-JNK (1 : 1000 dilution) were from Santa Cruz Biotechnology (Santa Cruz, CA, USA). Rabbit anti-p27 (1 : 1000 dilution), rabbit anti-p21 (1 : 1000 dilution), rabbit anti-cleaved caspase-3 (1 : 1000 dilution), rabbit anti-caspase-3 (1 : 1000 dilution), rabbit anti-caspase-6 (1 : 1000 dilution), rabbit anti-cleaved caspase-7 (1 : 1000 dilution), rabbit anti-caspase-7 (1 : 1000 dilution), rabbit anti-caspase-8 (1 : 1000 dilution), rabbit anti-caspase-9 (1 : 1000 dilution), rabbit anti-p21 (1 : 1000 dilution), rabbit anti-Bax (1 : 1000 dilution), rabbit anti-cleaved PARP1 (1 : 1000 dilution), rabbit anti-PAPR1 (1 : 1000 dilution), rabbit anti-Bcl2 (1 : 1000 dilution), and rabbit anti-BclXL (1 : 1000 dilution) antibodies were from Cell Signaling (Danvers, MA, USA). Goat anti-mouse and anti-rabbit horseradish peroxidase-conjugated IgG were obtained from Jackson ImmunoResearch (West Grove, PA, USA). ECL Western Blotting Detection Reagents were obtained from GeneDEPOT (Barker, TX, USA). Astemizole, ebastine, hydroxyebastine (HEB), and O-desmethylastemizole (DMA) were from Toronto Research Chemicals (North York, Canada). Pooled human liver microsomes (HLMs, H0630, and mixed gender) were obtained from XenoTech (Lenexa, KS, USA). Solvents were of high-performance liquid chromatography (HPLC) grade, and all other chemicals were of analytical grade.

### 2.2. Cell Culture

Human HCC Huh-7 and HepG2 cells were purchased from the Korean Cell Line Bank (Seoul, Korea). Both cells were incubated under standard conditions (37°C, 5% CO_2_, and 95% of humidity) in an incubator. Cells were maintained in RPMI 1640 (Thermo Fisher Scientific, Grand Island, NY, USA) supplemented with 10% heat-inactivated (56°C and 30 min) fetal bovine serum (FBS, Youngin, Seoul, Korea) and 1% penicillin/streptomycin antibiotics (Thermo Fisher Scientific, Grand Island, NY, USA). Each cell line was subcultured three times a week at a ratio of 1 : 2 or 1 : 3 dependent on confluency. Cell culture media were removed, and the dishes were washed twice with 5 mL phosphate-buffered saline (PBS). Cells were detached using 1 mL trypsin-EDTA solution (Gibco, Waltham, MA, USA) for 4 min. These were neutralized with 5 mL of FBS-containing media, and cells were collected by centrifugation for 3 min at 1350 rpm. Both cell lines were used at passages 5-20 for all experiments.

### 2.3. WST-1 Assays

Cells were seeded in 96-well culture plates at a density of 5 × 10^3^ cells per well and maintained for 24 h. After then, cells were treated with different concentrations of 6,8-diprenylorobol (0, 10, 20, 30, 40, 50, 60, and 70 *μ*M) and incubated for another 24, 48, and 72 h, respectively (total volume of each well is 200 *μ*L). After incubation, 10 *μ*L of EZ-Cytox (DoGenBio, Seoul, Korea) was added to each well and incubated for 1 h at 37°C. After incubation, the absorbance was measured at 450 nm using a spectrophotometer (Molecular Devices, Mountain View, CA, USA).

### 2.4. Detection of Morphological Change

Cells were seeded in 6-well culture plates at a density of 2 × 10^5^ cells per well, respectively. After 24 h of incubation, the cells were treated with different concentrations of 6,8-diprenylorobol (0, 20, 40, and 60 *μ*M) for 24 h. After treatment, morphological changes were observed and pictures were taken using microscopy (CKX53, Olympus, Shinjuku, Tokyo, Japan).

### 2.5. Cell Counting Assay

Cells were seeded in 6-well culture plates at a density of 2 × 10^5^ cells per well, respectively. After 24 h of incubation, cells were treated with different concentrations of 6,8-diprenylorobol (0, 20, 40, and 60 *μ*M) for 24 and 48 h. After treatment, media were removed and the cells were rinsed with 2 mL of PBS twice. The cells were detached using 350 *μ*L of trypsin-EDTA for 4 min and neutralized with 3 mL of FBS-containing media. The cells were collected by centrifugation for 5 min at 1500 rpm. The supernatant was removed, and the cells were suspended with 3 mL of media. The number of cells was counted using a hemocytometer.

### 2.6. Colony Formation Assay

Cells were seeded in 6-well culture plates at a density of 1 × 10^2^ cells per well, respectively. After 24 h of incubation, cells were treated with different concentrations of 6,8-diprenylorobol (0, 10, and 20 *μ*M) for 24 h. Then, media were replaced with the fresh media and incubated for 14 days. Cells were washed with PBS twice and stained with 1% crystal violet (Sigma) solution for 30 min.

### 2.7. Annexin V Staining Assay

The percentages of early and late apoptotic cells were measured using the FITC Annexin V apoptosis detection kit (BD Biosciences, Franklin Lakes, NJ, USA). Cells were seeded in six-well plates at a density of 2 × 10^5^ cells per well, respectively. After 24 h of incubation, cells were treated with different concentrations of 6,8-diprenylorobol (0, 20, 40, and 60 *μ*M) for 24 h and 48 h. After treatment, the media were removed and the cells were washed with 3 mL of PBS twice. The cells were detached using 350 *μ*L of trypsin-EDTA for 4 min and neutralized with 3 mL of FBS-containing media. The cells were collected by centrifugation for 5 min at 1500 rpm. The supernatant was removed, and the cells were suspended with 500 *μ*L of 1× binding buffer containing 5 *μ*L of annexin V and 5 *μ*L of propidium iodide (PI) for 15 min at room temperature in the dark. After staining, the cells were analyzed by flow cytometry (Beckman Coulter, Brea, CA, USA).

### 2.8. Cell Cycle Analysis

Cells were seeded in 6-well plates at a density of 2 × 10^5^ cells per well. After 24 h of incubation, cells were treated with different concentrations of 6,8-diprenylorobol (0, 20, 40, and 60 *μ*L) for 24 h and 48 h. After treatment, the media were removed and the cells were washed with 3 mL of PBS twice. Cells were detached using 350 *μ*L of trypsin-EDTA for 4 min and neutralized with 3 mL of FBS-containing media. Cells were collected by centrifugation for 5 min at 1500 rpm. After removing the supernatant, cells were stained with PI working solution containing RNase A and 0.2% Triton X-100 (50 *μ*g/mL PI and 200 *μ*g/mL RNase A) for 30 min at 37°C. Cell cycle distribution analysis was conducted using flow cytometry (Beckman Coulter, Brea, CA, USA).

### 2.9. Terminal Deoxynucleotidyl Transferase- (TdT-) Mediated dUTP Nick-End Labeling (TUNEL) Assay

The fluorometric TUNEL detection system was purchased from Promega (Madison, WI, USA). Cells were seeded in 6-well plates at a density of 2 × 10^5^ cells per well and incubated for 24 h under standard conditions. After incubation, cells were treated with different concentrations of 6,8-diprenlyorobol (0, 20, and 40 *μ*M) for 24 and 48 h, respectively. After treatment, cells were fixed with 6% of formaldehyde for 25 min at room temperature and permeabilized using 0.2% of Triton X-100 for 5 min at room temperature. After then, cells were incubated with 50 *μ*L of TdT enzyme buffer for 1 h at 37°C. The cell nucleus was stained with Hoechst staining solution (Sigma). 10 *μ*L of Hoechst staining solution was dissolved in 10 mL of PBS. Labeled strand breaks were detected using fluorescence microscopy (CKX53, Olympus, Shinjuku, Tokyo, Japan).

### 2.10. CYP2J2 Activity Assays

All incubations were performed in triplicate, and data are presented as average values. The inhibitory potential of 6,8-diprenylorobol against CYP2J2-mediated astemizole *O*-demethylation and ebastine hydroxylase activity was determined using pooled human liver microsomes (HLMs) in the absence and presence of the test compound. In brief, the incubation mixtures (final volume, 100 *μ*L) containing pooled HLMs (0.25 mg/mL), CYP2J2 probe substrate (astemizole or ebastine), and 6,8-diprenylorobol were preincubated for 5 min at 37°C. The reaction was initiated by the addition of a NADPH-generating system (1.3 mM NADP^+^, 3.3 mM glucose-6-phosphate, 3.3 mM MgCl_2_, and 500 units/mL glucose-6-phosphate dehydrogenase) after preincubation. To determine the inhibitory potentials (*K*_*i*_ values) of 6,8-diprenylorobol for CYP2J2-mediated astemizole *O*-demethylation and ebastine hydroxylation in HLMs, 6,8-diprenylorobol (0, 2.5, 5, 10, and 20 *μ*M for astemizole and 0, 0.5, 2, 4, and 6 *μ*M for ebastine) was added to reaction mixtures containing different concentrations of astemizole (1 and 5 *μ*M) or ebastine (0.2 and 0.5 *μ*M). After preincubation at 37°C, the reactions were maintained for 20 min in a thermoshaker. The reactions were terminated by the addition of 100 *μ*L of ice-cold acetonitrile containing 10 nM trimipramine (internal standard, IS) into the mixtures. After mixing and centrifuging at 13,000 g for 5 min at 4°C, aliquots of the supernatants were analyzed by liquid chromatography-tandem mass spectrometry (LC-MS/MS).

### 2.11. siRNA Transfection

siRNA against CYP2J2 and control siRNA were purchased from Santa Cruz Biotechnology. For transfection with siRNA, cells were transfected with CYP2J2 siRNA or control siRNA using the Lipofectamine 2000 transfection reagent (Thermo Scientific, Rockford, IL, USA) according to the manufacturer's protocol.

### 2.12. ROS Detection by Flow Cytometry

Intracellular ROS level was measured using the stable nonpolar dye DCF-DA, which readily diffuses into the cells. Cells were treated with either 20 (Huh-7) and 60 (HepG2) *μ*M of 6,8-diprenylorobol, 10 mM of NAC, or 20 (Huh-7) and 60 (HepG2) *μ*M of 6,8-diprenylorobol+10 mM of NAC for 24 h and then incubated at 37°C with 20 *μ*M of DCF-DA for 30 min. After incubation, the ROS level was measured by flow cytometry (Beckman Coulter).

### 2.13. Statistical Analysis

Results are expressed as arithmetic mean ± SEM (the standard error of the mean). To compare the statistical meaning between the groups, two-sided unpaired Student's *t*-test was used. All experiments were repeated three times, and the representative data were shown. Statistical analyses were performed using SPSS software (version 19.0, SPSS Inc., Chicago, IL, USA). Mean differences with *p* values less than 0.05 were considered statistically significant.

## 3. Results

### 3.1. 6,8-Diprenylorobol Inhibits the Proliferation of Huh-7 and HepG2 Cells

**Figure 1:**
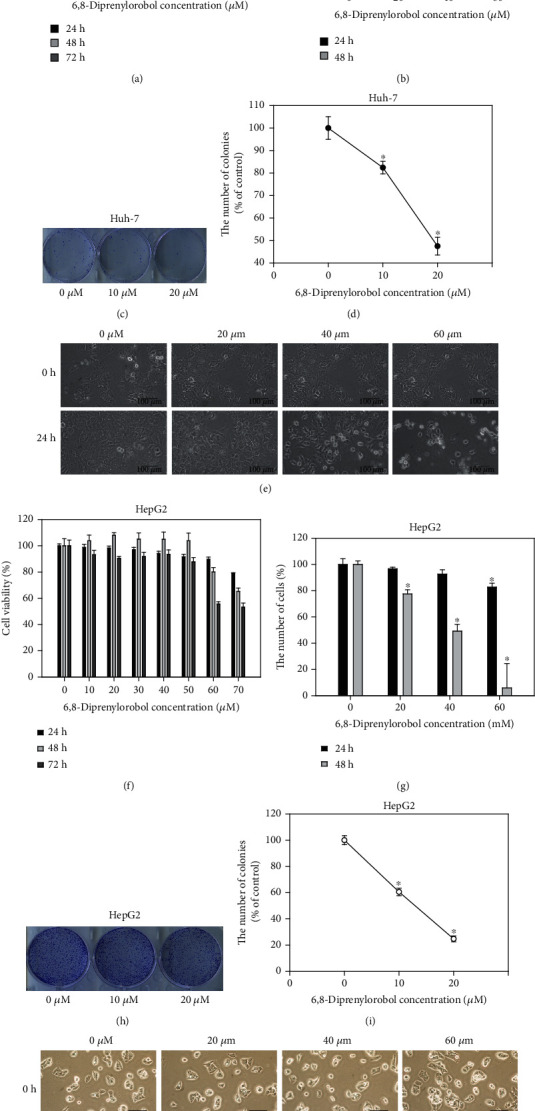
Cytotoxic and antiproliferative effect of 6,8-diprenylorobol against (a) Huh-7 and (b) HepG2 cells. Dose-dependent effect of 6,8-diprenylorobol (0, 10, 20, 30, 40, 50, 60, and 70 *μ*M) against Huh-7 and HepG2 cells after 24, 48, and 72 h incubation. The cell viability was determined by the WST-1 assay. Cell counting assay of Huh-7 and HepG2 cells treated with 6,8-diprenylorobol (0, 20, 40, and 60 *μ*M) for 24 and 48 h. Morphological changes of Huh-7 and HepG2 cells treated with 6,8-diprenylorobol. Colony formation assay of Huh-7 and HepG2 cells treated with 6,8-diprenylorobol (0, 10, and 20 *μ*M) for 14 days. This result is one of the representative data from three biological replicates, and the error bars mean STE. ∗ means *p* value < 0.05.

To investigate the antiproliferative effect of 6,8-diprenylorobol, Huh-7 and HepG2 cells were treated with the indicated dose of 6,8-diprenylorobol. As shown in Figures 1(a) and 1(b), the viability of Huh-7 and HepG2 cells was decreased after 6,8-diprenylorobol treatment. The cell viability of Huh-7 cells treated with 0, 10, 20, 30, 40, 50, 60, and 70 *μ*M of 6,8-diprenylorobol was 100, 106, 95, 87, 66, 53, 48, and 46% for 24 h, 100, 99, 103, 94, 70, 40, 35, and 34% for 48 h, and 100, 124, 115, 105, 61, 36, 35, and 36% for 72 h, respectively. The cell viability of HepG2 cells treated with 0, 10, 20, 30, 40, 50, 60, and 70 *μ*M of 6,8-diprenylorobol was 100, 99, 98, 97, 94, 92, 90, and 79% for 24 h, 100, 104, 108, 105, 105, 104, 80, and 65% for 48 h, and 100, 93, 91, 92, 93, 88, 56, and 53% for 72 h, respectively. Furthermore, cell counting assay results indicated that the number of colonies decreased significantly after treatment with 6,8-diprenylorobol. The number of colonies decreased to 82% and 47% in Huh-7 cells and 60% and 24% in HepG2 cells after treatment with 10 and 20 *μ*M of 6,8-diprenylorobol for 14 days. Additionally, we observed the detectable morphological changes of Huh-7 and HepG2 cells treated with 6,8-diprenylorobol for 24 h. Taken together, our results demonstrated that 6,8-diprenylorobol has an antiproliferative effect against Huh-7 and HepG2 cells.

### 3.2. 6,8-Diprenylorobol Induces Cell Cycle Arrest in Huh-7 and HepG2 Cells

**Figure 2:**
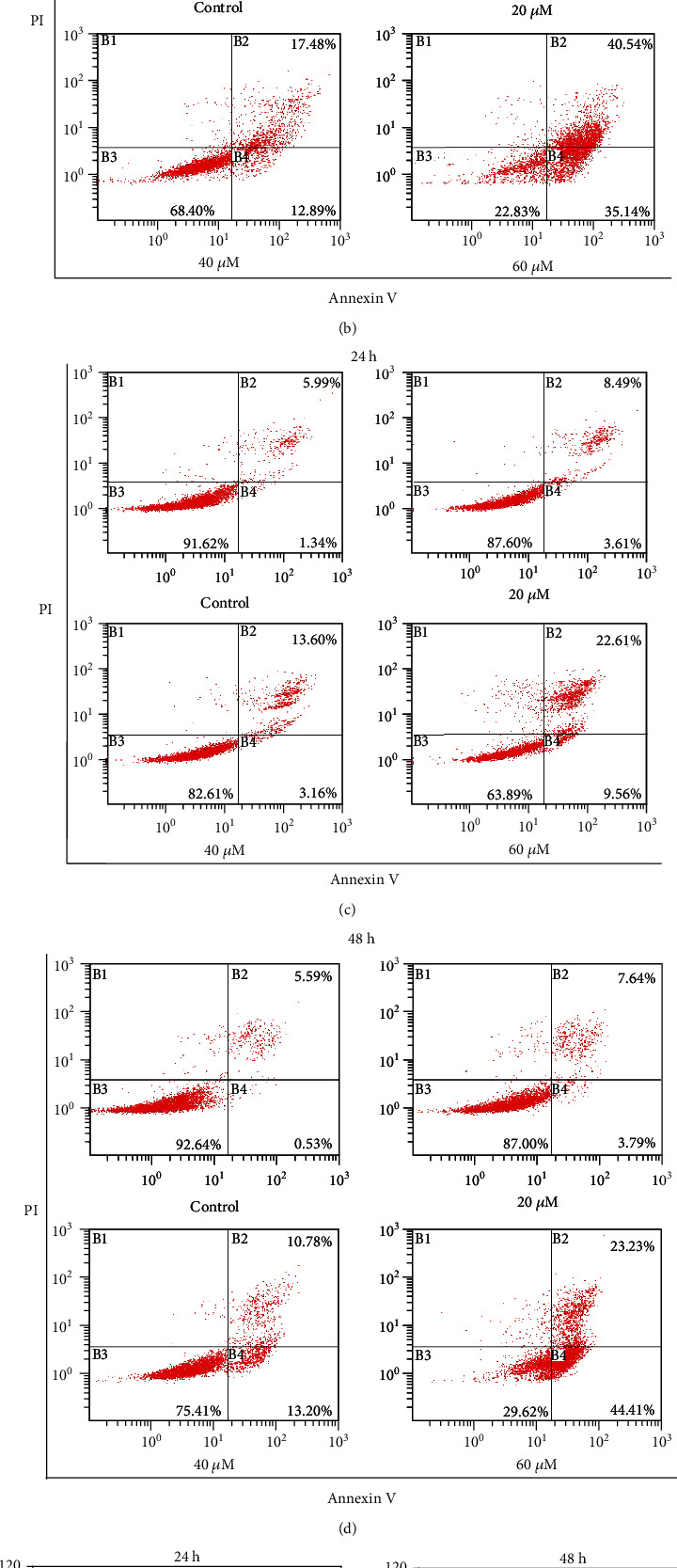
Cell cycle analysis of (a) Huh-7 and (b) HepG2 cells after 24 and 48 h of treatment with different concentrations of 6,8-diprenylorobol. Huh-7 and HepG2 cells were treated with 0, 20, and 40 *μ*M of 6,8-diprenylorobol for 24 and 48 h and stained with PI. After staining, the cells were analyzed by flow cytometry. The distribution and percentage of cells in G0/1, S, and G2/M phases of the cell cycle were shown for Huh-7 and HepG2. This result is one of the representative data from three biological replicates, and the error bars mean STE. ∗ means *p* value < 0.05.

To evaluate the effects of 6,8-diprenylorobol on the cell cycle, Huh-7 and HepG2 cells were treated with different concentrations of 6,8-diprenylorobol (0, 20, and 40 *μ*M) for 24 and 48 h and the cell population was analyzed by flow cytometry. As shown in Figures 2(a) and 2(b), cell cycle analysis results indicated that the percentage of cells in the G0/1 population was increased after treatment with 6,8-diprenylorobol compared to that of the control group. The G0/1 population of Huh-7 cells treated with 0, 20, and 40 *μ*M of 6,8-diprenylorobol for 24 and 48 h was 46, 55, and 62% and 53, 65, and 75%, respectively. The G0/1 population of HepG2 cells treated with 0, 20, and 40 *μ*M of 6,8-diprenylorobol for 24 h and 48 h was 36, 37, and 40% and 44, 47, and 58%, respectively. Statistical analysis indicated that the G0/1 population of Huh-7 cells was increased significantly (*p* < 0.05) after treatment with 20 and 40 *μ*M of 6,8-diprenylorobol for 24 h and 48 h. Additionally, the G0/1 population of HepG2 cells was increased significantly (*p* < 0.05) after treatment with 40 *μ*M of 6,8-diprenylorobol for 24 h and 48 h. Taken together, our results demonstrated that 6,8-diprenylorobol treatment induced G0/1 phase cell cycle arrest in Huh-7 and HepG2 cells.

### 3.3. 6,8-Diprenylorobol Induces Apoptosis in Huh-7 and HepG2 Cells

**Figure 3:**
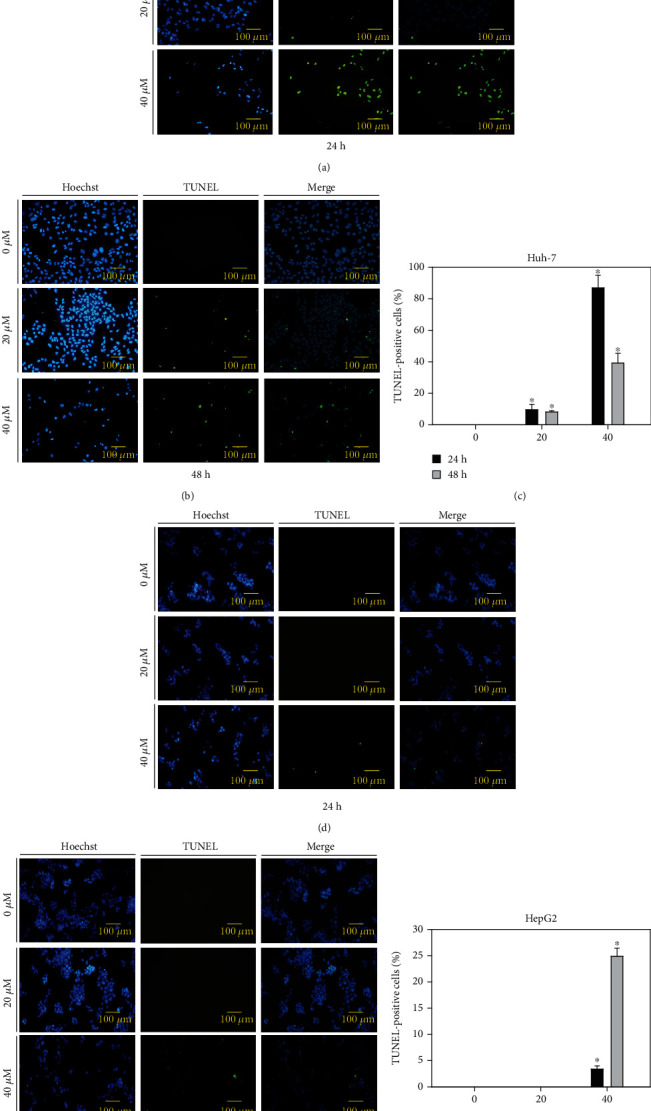
Evaluation of apoptosis in Huh-7 and HepG2 cells by the annexin V/PI double staining assay after 24 and 48 h of treatment with different concentrations of 6,8-diprenylorobol. The percentages of apoptotic cells (upper right and lower right) are shown (B1: necrotic cells, B2: late apoptotic cells, B3: survival cells, and B4: early apoptotic cells). (a) Huh-7 flow cytometry dot plots. (b) HepG2 flow cytometry dot plots. This result is one of the representative data from three biological replicates, and the error bars mean STE. ∗ means *p* value < 0.05.

To investigate 6,8-diprenylorobol-induced apoptosis in Huh-7 and HepG2 cells, we conducted an annexin V/PI double staining assay. Huh-7 and HepG2 cells were stained with annexin V and PI dye after various concentrations of 6,8-diprenylorobol (0, 20, 40, and 60 *μ*M) treatment for 24 and 48 h. After treatment, cells were analyzed by flow cytometry. Annexin V/PI double staining assay results showed that annexin V^+^/PI^−^ cells (early apoptosis) and annexin V^+^/PI^+^ cells (late apoptosis) were increased after treatment with 6,8-diprenylorobol in a dose- and time-dependent manner (Figures 3(a) and 3(b)). The total apoptotic cell rate (early+late apoptosis) of Huh-7 cells treated with 0, 20, 40, and 60 *μ*M of 6,8-diprenylorobol was 8.16, 16.43, 19.18, and 41.61% for 24 h and 10.54, 24.39, 30.37, and 75.68% for 48 h, respectively. The total apoptotic cell rate of HepG2 cells treated with 0, 20, 40, and 60 *μ*M of 6,8-diprenylorobol was 7.33, 12.10, 16.76, and 32.17% for 24 h and 6.12, 11.43, 23.98, and 67.64% for 48 h, respectively. These results showed that 6,8-diprenylorobol treatment induced apoptosis in Huh-7 and HepG2 cells.

### 3.4. 6,8-Diprenylorobol Induces DNA Fragmentation in Huh-7 and HepG2 Cells

**Figure 4:**
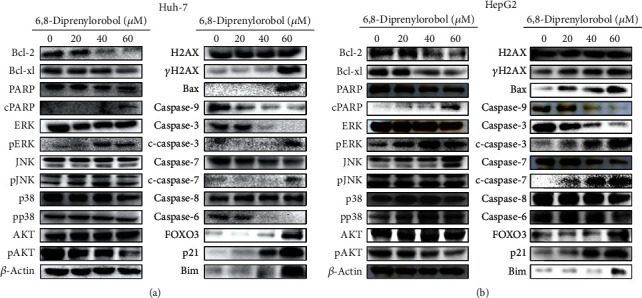
Detection of DNA fragmentation in apoptotic cells using the TUNEL assay in (a) Huh-7 and (b) HepG2 cells. Huh-7 and HepG2 cells were treated with 0, 20, and 40 *μ*M of 6,8-diprenylorobol for 24 and 48 h, and induction of DNA fragmentation was visualized by fluorescence microscopy (100 × 100). Blue fluorescence shows the nuclei stained with Hoechst, and green fluorescence shows fragmented DNA stained with TUNEL indicating DNA fragmentation. The merged images represent the merging of blue stained nuclei with green stained nick label.

To detect 6,8-diprenylorobol-induced DNA fragmentation in Huh-7 and HepG2 cells, we performed the TUNEL assay. Huh-7 and HepG2 cells were treated with various concentrations of 6,8-diprenylorobol (0, 20, and 40 *μ*M) for 24 and 48 h, and DNA fragmentation in nuclei was detected by fluorescence microscopy. TUNEL assay results showed that green fluorescence (damaged DNA) was increased after 6,8-diprenylorobol treatment. Hoechst staining was performed to stain nuclei (Figures 4(a) and 4(b)). Merged images represented the fragmentation of DNA in nuclei of Huh-7 and HepG2 cells treated with 6,8-diprenylorobol. The percentage of TUNEL-positive cells of Huh-7 after treatment with 0, 20, and 40 *μ*M of 6,8-diprenylorobol was 0, 9.68, and 87% for 24 h and 0, 8.1, and 39.2% for 48 h, respectively. The percentage of TUNEL-positive cells of HepG2 after treatment with 0, 20, and 40 *μ*M of 6,8-diprenylorobol was 0, 0, and 3.4% for 24 h and 0, 0, and 24.9% for 48 h, respectively. These results showed that 6,8-diprenylorobol treatment causes DNA fragmentation of nuclei, one property of apoptotic cells, in Huh-7 and HepG2 cells.

### 3.5. 6,8-Diprenylorobol Activates the Apoptotic Signaling Pathway in Huh-7 and HepG2 Cells

**Figure 5:**
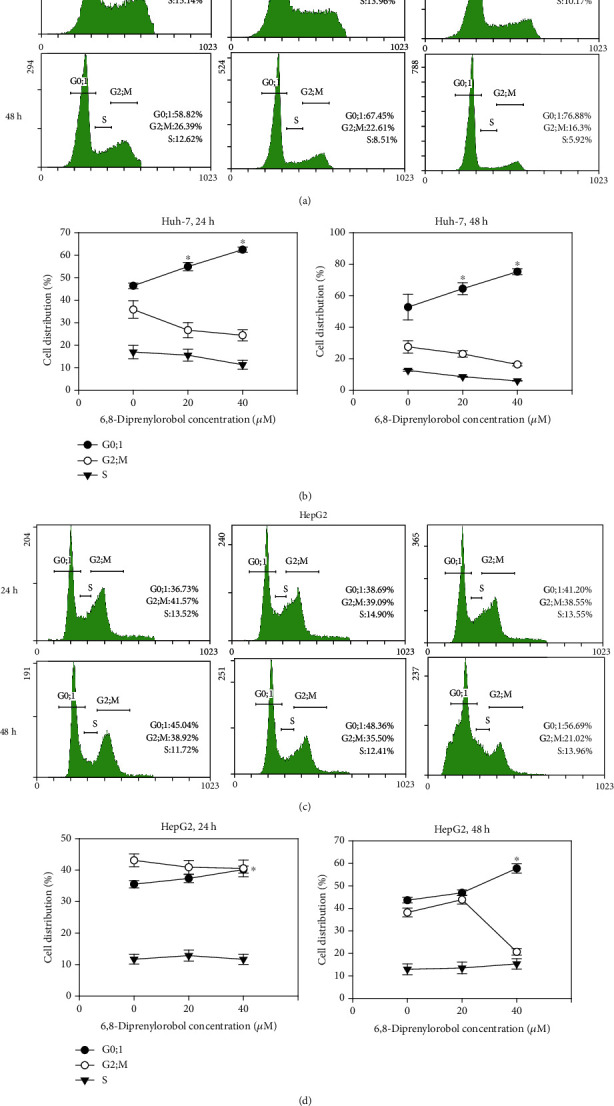
Western blot analysis of (a) Huh-7 and (b) HepG2 cells by the treatment with 6,8-diprenylorobol. Cells were treated with different concentrations of 6,8-diprenylorobol (0, 20, 40, and 60 *μ*M) for 24 h, and western blot was performed to measure protein expression level using specific antibodies. *β*-Actin was used for a gel loading control.

**Figure 6:**
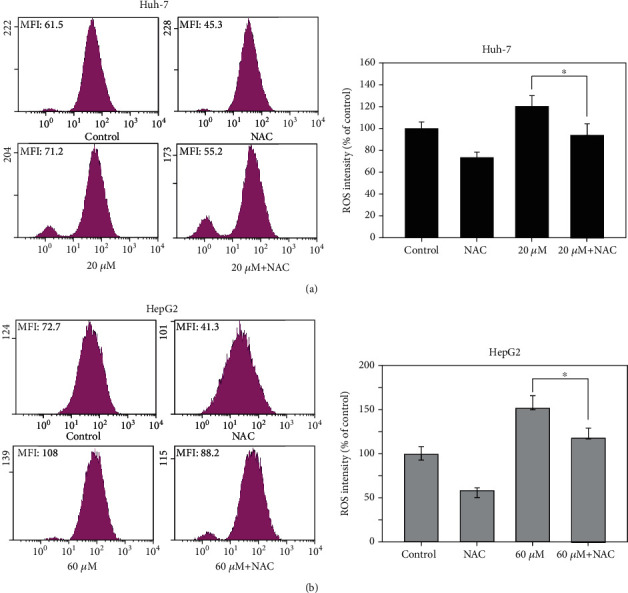
Measurement of ROS level in (a) Huh-7 and (b) HepG2 cells after treatment with 6,8-diprenylorobol. Cells were treated with 10 mM of NAC and/or 20 (Huh-7) and 60 (HepG2) *μ*M of 6,8-diprenylorobol for 24 h, and DCF-DA staining was performed to measure fluorescence intensity. This result is one of the representative data from three biological replicates, and the error bars mean STE. ∗ means *p* value < 0.05.

To investigate 6,8-diprenylorobol-induced apoptotic signaling pathways in Huh-7 and HepG2 cells, we performed western blotting. We analyzed the expression levels of proteins associated with cell survival and apoptosis pathway after treatment with 6,8-diprenylorobol (0, 20, 40, and 60 *μ*M) for 24 h. As shown in Figures 5(a) and 5(b), we found the increased expression level of cleaved PARP1, cleaved caspase-3, cleaved caspase-7, FOXO3, Bax, Bim, p21, and p27 but decreased expression level of Bcl2, BclXL, caspase-6, and caspase-9 in a dose-dependent manner. In addition, we analyzed the phosphorylation status of kinases related to cell survival and apoptosis pathway after treatment with 6,8-diprenylorobol. As shown in Figures 5(a) and 5(b), 6,8-diprenylorobol treatment decreased the level of pAKT but increased the level of pERK in a dose-dependent manner. Taken together, our results showed that 6,8-diprenylorobol treatment activated the intrinsic apoptotic signaling pathways and regulated AKT and ERK in HepG2 and Huh7 cells. Since FOXO3 could be regulated by DNA damage in cells, we tried to investigate whether the level of ROS was increased by 6,8-diprenylorobol treatment or not. As shown in Figures 6(a) and 6(b), flow cytometry results showed that the MFI values of the control, NAC, 6,8-diprenylorobol, and NAC+6,8-diprenylorobol treatment were 61.5, 45.3, 71.2, and 55.2 in Huh-7 and 72.7, 41.3, 108, and 88.2 in HepG2, respectively. This result suggests that 6,8-diprenylorobol treatment induced the increase in ROS level in cells, which might be related to DNA damage in cells triggering the apoptotic signaling pathway.

### 3.6. 6,8-Diprenylorobol Inhibits CYP2J2 Activity

**Figure 7:**
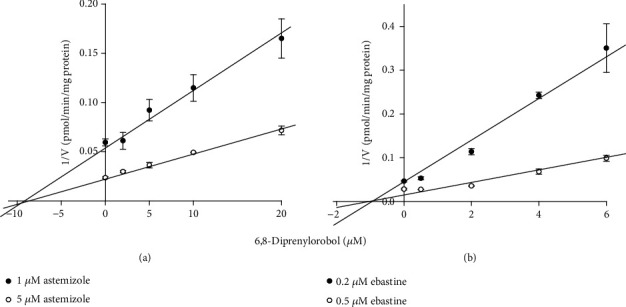
Dixon plots for inhibition of CYP2J2-catalyzed (a) astemizole *O*-demethylation and (b) ebastine hydroxylation by 6,8-diprenylorobol in pooled HLMs. An increasing concentration of (a) astemizole (2 and 5 *μ*M) or (b) ebastine (0.2 and 0.5 *μ*M) was incubated with HLMs (0.25 mg/mL, XenoTech H0630) and an NADPH-generating system at 37°C for 20 min in the presence or absence of 6,8-diprenylorobol. This result is one of the representative data from three biological replicates, and the error bars mean STE.

To elucidate the mechanistic target for the anticancer activity of 6,8-diprenylorobol, we evaluated the inhibitory potential of 6,8-diprenylorobol against the CYP2J2 enzyme using HLMs. 6,8-Diprenylorobol inhibited CYP2J2-mediated astemizole *O*-demethylase activity with an IC_50_ value of 7.33 *μ*M. To further investigate the mechanism of CYP2J2 inhibition by 6,8-diprenylorobol in HLMs, a kinetic study of CYP2J2-mediated astemizole *O*-demethylase and ebastine hydroxylase activities in the presence of 6,8-diprenylorobol was performed. Dixon and Lineweaver-Burk plots indicated that 6,8-diprenylorobol inhibited CYP2J2 enzyme activity with an apparent *K*_*i*_ value of 9.46 and 2.61 *μ*M on CYP2J2-mediated astemizole *O*-demethylase and ebastine hydroxylase activities, respectively. In addition, 6,8-diprenylorobol noncompetitively inhibited CYP2J2-mediated astemizole *O*-demethylase and ebastine hydroxylase activity (Figures 7(a) and 7(b)).

### 3.7. CYP2J2 siRNA Transfection Enhances the Anticancer Effect of 6,8-Diprenylorobol in Huh-7 and HepG2 Cells

**Figure 8:**
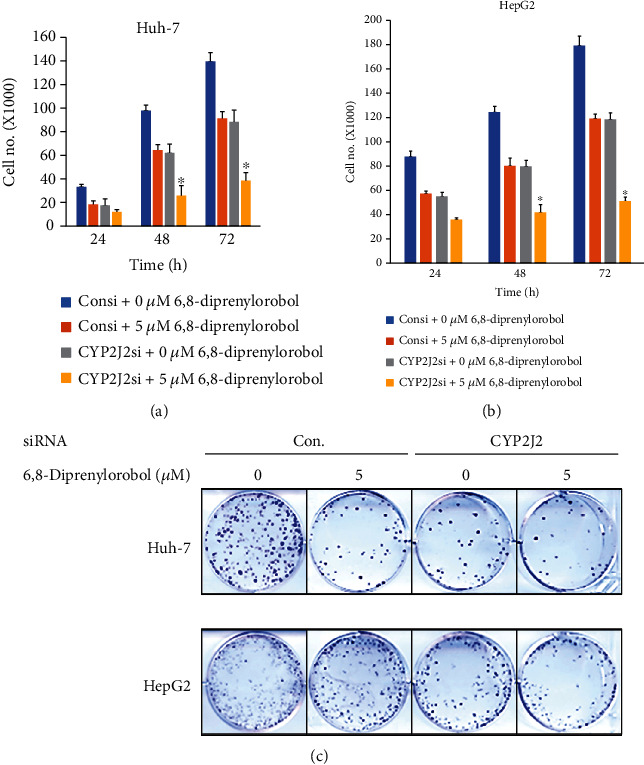
Cytotoxic and antiproliferative effect of the combination of CYP2J2 siRNA transfection and 6,8-diprenylorobol treatment in Huh-7 and HepG2 cells. Huh-7 and HepG2 cells were transfected with the control or siRNA against CYP2J2 followed by treatment with 6,8-diprenylorobol (0 or 5 *μ*M) for 24, 48, and 72 h for WST-1 and cell counting assays and 14 days for the colony formation assay. This result is one of the representative data from three biological replicates, and the error bars mean STE. ∗ means *p* value < 0.05.

**Figure 9:**
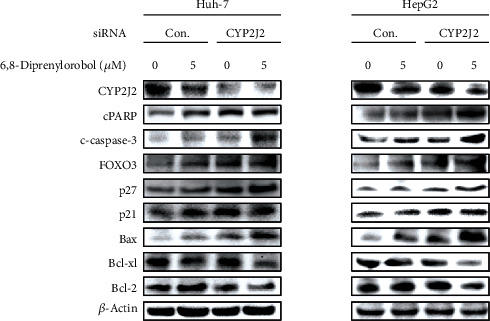
Western blot analysis of Huh-7 and HepG2 cells transfected with control or CYP2J2 siRNA followed by the treatment with 6,8-diprenylorobol (0 and 5 *μ*M) for 24 h on survival and apoptosis-related proteins as indicated. *β*-Actin is used for a gel loading control.

To investigate the combined anticancer effect of CYP2J2 siRNA transfection and 6,8-diprenylorobol treatment in Huh-7 and HepG2 cells, we transfected Huh-7 and HepG2 cells with the control or CYP2J2 siRNA and treated cells with 5 *μ*M of 6,8-diprenylorobol that was not an effective dose. As shown in Figure 8, treatment with 6,8-diprenylorobol in Huh-7 and HepG2 cells after transfection with CYP2J2 siRNA enhanced the cytotoxicity and antiproliferative effect of 6,8-diprenylorobol. Then, we analyzed the expression of proteins related to apoptosis in Huh-7 and HepG2 cells. As shown in Figure 9, treatment with 6,8-diprenylorobol after transfection with CYP2J2 siRNA decreased synergistically the Bcl2 and BclXL expression but increased synergistically the cleaved PARP1, cleaved caspase-3, FOXO3, Bax, p27, and p21 expression in Huh-7 and HepG2 cells. Thus, these results indicated that downregulation of CYP2J2 sensitized Huh-7 and HepG2 cells to 6,8-diprenylorobol treatment and CYP2J2 is associated with apoptosis in Huh-7 and HepG2 cells treated with 6,8-diprenylorobol.

## 4. Discussion

Cancer is one of the leading causes of death in the United States as well as globally [36, 37]. Recently, drug-targeted therapies have been developed and improved cancer patient care [38]. However, there are still several side effects of the current cancer therapies on the advanced metastasized cancer [39, 40]. Therefore, searching for a more effective and less dangerous treatment is required to improve the efficiency of treatment and reduce the treatment cost for cancer care. Recently, cancer chemoprevention with natural phytochemicals has been recognized as a promising strategy to prevent and treat cancer [41].

As a natural compound, we expect that 6,8-diprenylorobol plays a crucial role in cancer therapy. For the last fifteen years, several studies showed that 6,8-diprenylorobol has an anti-*Helicobacter pylori* effect and antiestrogenic activity [34, 35]. However, the potential effects of 6,8-diprenylorobol on various diseases have not been investigated well. Specifically, there is only one study investigating the anticancer effect of 6,8-diprenylorobol on cancer cells [42]. According to them, 6,8-diprenylorobol showed potent cytotoxic effects toward HL-60 human leukemia cells with an IC_50_ value of about 10 *μ*M. Although it was not a direct study to show the anticancer activity of 6,8-diprenylorobol, Sun et al. reported that 6,8-diprenylorobol inhibited aromatase, one of the targetable enzymes for cancer therapy, with a *K*_*i*_ value of 1.42 *μ*M [43]. In this study, we focus on studying the effect of 6,8-diprenylorobol on human HCC Huh-7 and HepG2 cells.

Cell cycle arrest means that cells are no longer involved in the duplication and division process [44]. Many studies showed that lots of phytochemicals could induce cell cycle arrest in various cancer cell lines. Phytochemical extracts from cranberry induce G0/1 phase cell cycle arrest and apoptosis in human breast cancer MCF-7 cells [45]. Furthermore, gallic acid induces G0/1 phase cell cycle arrest and apoptosis through inhibition of cyclins D and E and activating a mitochondria-dependent apoptotic pathway in human leukemia HL-60 cells [46]. As shown in Figures 2(a) and 2(b), our results suggested that 6,8-diprenylorobol induced G0/1 cell cycle arrest in Huh-7 and HepG2 cells.

Previously, we reported that CYP2J2 downregulation by siRNA transfection combined with acetylshikonin or broussochalcone A treatment induced apoptosis in HCC cells via activation of FOXO3 and inhibition of CYP2J2 [47, 48]. In this study, we observed that 6,8-diprenylorobol showed the similar results with our previous report. To identify which kinase is involved in 6,8-diprenylorobol-mediated activation of FOXO3 and inhibition of CYP2J2, we analyzed the phosphorylated status of AKT, ERK, JNK, and p38. FOXO3 has been known to participate in cell growth inhibition by upregulating cell cycle regulation and proapoptotic proteins transcriptionally [49]. As shown in Figure 5, the phosphorylated AKT was significantly decreased by 6,8-diprenylorobol treatment, but the phosphorylated ERK was significantly increased by 6,8-diprenylorobol treatment. The phosphorylation of JNK and p38 was not critically changed. FOXO3 could be phosphorylated in Thr32, Ser253, and Ser315 by AKT, leading to faster protein degradation [49]. Thus, the inhibition of the phosphorylation of AKT by 6,8-diprenylorobol might contribute to the enhancement of the tumor-suppressive transcriptional activity of FOXO3 to block Huh-7 and HepG2 cell growth.

The ERK cascade is one of the major signaling pathways of the mitogen-activated protein kinase (MAPK) signaling, and it plays a crucial role in the regulation of cell proliferation, differentiation and cell cycle, and apoptosis [50, 51]. According to a previous research, ERK activation induced cell cycle arrest and DNA damage-induced apoptosis [52]. Furthermore, it is reported that activation of ERK plays an important role in quercetin-induced apoptosis in lung carcinoma A549 cells [53]. ERK is required for the activation of cisplatin-induced apoptosis by mediating the mitochondria-dependent apoptotic signaling in renal epithelial cells [54]. As shown in Figures 5(a) and 5(b), our results showed that 6,8-diprenylorobol activated ERK and it might modulate apoptotic signaling pathways in Huh-7 and HepG2 cells, which was consistent with the previous references.

CYP2J2 is an epoxygenase enzyme, and its role is to metabolize arachidonic acid to epoxyeicosatrienoic acids [55]. It is reported that CYP2J2 is highly upregulated in various human carcinoma cell lines and CYP2J2 could promote human cancer metastasis and tumor cell growth [56, 57]. According to a previous study, the doxorubicin-induced reduction of viability was markedly attenuated by upregulation of CYP2J2 expression. The increase in the Bax/Bcl2 ratio and the decrease in procaspase-3 expression level were also recovered by CYP2J2 upregulation [20]. Therefore, CYP2J2 may be an important target to develop the effective therapeutic methods for cancers. In fact, we have reported that acetylshikonin or broussochalcone A has anticancer activity in HCC cells through the inhibition of CYP2J2 [47, 48]. We observed that 6,8-diprenylorobol inhibited CYP2J2-mediated astemizole *O*-demethylase and ebastine hydroxylase activity with an IC_50_ value of 7.33 *μ*M, which is comparable to the IC_50_ values of decursin (IC_50_ = 6.95 *μ*M) [58], thelephoric acid (IC_50_ = 3.23 *μ*M) [59], and tanshinone IIA (IC_50_ = 2.5 *μ*M) [60], in a noncompetitive way. The inhibitory potential of 6,8-diprenylorobol was less potent than those of danazol (*K*_*i*_ = 0.06 *μ*M) [61] and hydroxyebastine (*K*_*i*_ = 0.45 *μ*M) [62], while it was similar or more potent than that of decursin (*K*_*i*_ = 8.34 *μ*M) [58].

In conclusion, 6,8-diprenylorobol showed anticancer activity against HCC Huh-7 and HepG2 cells. We think that this anticancer activity of 6,8-diprenylorobol might result from G0/1 cell cycle arrest and upregulation of proapoptotic proteins via activation of FOXO3 in Huh-7 and HepG2 cells. Also, we found that 6,8-diprenylorobol has inhibitory activity against CYP2J2 in a noncompetitive manner, which could be associated with the anticancer activity of 6,8-diprenylorobol in Huh-7 and HepG2 cells. For further study, we are currently planning to investigate the detailed anticancer working mechanisms of 6,8-diprenylorobol and perform xenograft mouse experiments.

## Data Availability

The authors confirm that the data supporting the findings of this study are available within the article.

## Abbreviation

HCC: Human hepatocellular carcinoma
CYP2J2: Cytochrome P450 2J2
TUNEL: Terminal deoxynucleotidyl transferase dUTP nick-end labeling
PARP: Poly (ADP-ribose) polymerase
cPARP: Cleaved poly (ADP-ribose) polymerase
FOXO3: Forkhead box O3
DMSO: Dimethyl sulfoxide
FBS: Fetal bovine serum
PI: Propidium iodide
ROS: Reactive oxygen species.
